# Effects of an oral synbiotic on the gastrointestinal immune system and microbiota in patients with diarrhea-predominant irritable bowel syndrome

**DOI:** 10.1007/s00394-018-1826-7

**Published:** 2018-09-24

**Authors:** Adrian Mathias Moser, Walter Spindelboeck, Bettina Halwachs, Heimo Strohmaier, Patrizia Kump, Gregor Gorkiewicz, Christoph Högenauer

**Affiliations:** 1 Division of Gastroenterology and Hepatology, Department of Internal Medicine, Medical University of Graz, Auenbruggerplatz 15, 8036 Graz, Austria; 2 Theodor Escherich Laboratory for Microbiome Research, Medical University of Graz, Auenbruggerplatz 15, 8036 Graz, Austria; 3 Institute of Pathology, Medical University of Graz, Auenbruggerplatz 25, 8036 Graz, Austria; 4 Center for Medical Research, Medical University of Graz, Stiftingtalstraße 24, 8010 Graz, Austria

**Keywords:** Irritable bowel syndrome, Microbiota, Short-chain fatty acids, Mucosal immune cells

## Abstract

**Purpose:**

Diarrhea-predominant irritable bowel syndrome (IBS-D) is a common functional gastrointestinal disorder. Probiotics and synbiotics have been shown to improve symptoms of IBS, although mechanisms of action are currently not understood.

**Methods:**

We investigated the effects of a 4-week oral synbiotic treatment (OMNi-BiOTiC^®^ Stress Repair) in ten IBS-D patients on gastrointestinal mucosal and fecal microbiota, mucosa-associated immune cells, and fecal short-chain fatty acids. The upper and lower gastrointestinal tracts were compared before and after a 4-week synbiotic treatment using endoscopic evaluation to collect mucosal specimens for FACS analysis and mucosal 16S rRNA gene analysis. In stool samples, analysis for fecal SCFAs using GC–MS, fecal zonulin using ELISA, and fecal 16S rRNA gene analysis was performed.

**Results:**

Synbiotics led to an increased microbial diversity in gastric (*p* = 0.008) and duodenal (*p* = 0.025) mucosal specimens. FACS analysis of mucosal immune cells showed a treatment-induced reduction of CD4^+^ T cells (60 vs. 55%, *p* = 0.042) in the ascending colon. Short-chain fatty acids (acetate 101 vs. 202 µmol/g; *p* = 0.007) and butyrate (27 vs. 40 µmol/g; *p* = 0.037) were elevated in fecal samples after treatment. Furthermore, treatment was accompanied by a reduction of fecal zonulin concentration (67 vs. 36 ng/ml; *p* = 0.035) and disease severity measured by IBS-SSS (237 vs. 54; *p* = 0.002).

**Conclusions:**

Our findings indicate that a short-course oral synbiotic trial may influence the human gastrointestinal tract in IBS-D patients on different levels which are region specific.

**Electronic supplementary material:**

The online version of this article (10.1007/s00394-018-1826-7) contains supplementary material, which is available to authorized users.

## Introduction

Irritable bowel syndrome (IBS) is a functional disorder of the gastrointestinal (GI) tract. Since the global prevalence of IBS is estimated to be between 10 and 20% depending on diagnostic criteria and geographic region, it constitutes a major medical burden and is thereby considered a public health issue [1]. The subtype diarrhea-predominant IBS (IBS-D) reflects diarrhea as the predominant symptom. Diagnosis is made by the symptom-based ROME criteria after the exclusion of organic disease [2]. To date, the treatment of IBS-D is focused on relief of symptoms rather than cure and consists of nutritional interventions, psychological or medical therapies [3].

Intestinal mucosal inflammation is a hallmark of disease in IBS-D [4, 5]. Specific compositional changes in the microbiota are thought to trigger these mucosal inflammatory responses in IBS-D which in turn are able to stimulate visceral hypersensitivity and pain [3, 4, 6, 7]. Thereby, the possible dysbiosis-triggered mucosal inflammation involves elevated pro-inflammatory cytokines that can interact with colonic nociceptive and non-nociceptive afferent nerves. If activated, these afferent nerves can sensitize mechanosensory colonic c-fibers to mediate pain symptoms in IBS-D patients [4]. Furthermore, activated mast, B and plasma cells accompanied with an impaired barrier function in jejunal specimens are present in IBS-D patients and the grade of mucosal inflammation is thereby associated to clinical disease activity. These microbiota-neuro-immunological interactions might, therefore, be involved in triggering intestinal hypersensitivity and pain, suggesting that IBS-D has a microbiota-dependent immune-mediated pathogenesis [8–10].

Therapies for IBS-D influencing the composition and function of the intestinal ecosystem are, therefore, of interest although mechanisms of action of probiotic (preparations with microorganisms only), prebiotic (food ingredients that enhance the growth of specific microorganism, e.g., inulin, starch), and synbiotic (combination of pre-and probiotics) therapies are not fully understood. However, extensive research is ongoing to clarify the role of specific probiotic strains and formulations on the host [11–13]. A recent meta-analysis including several oral probiotics in IBS suggests a clinical efficacy with significant decrease in various IBS-related symptoms after treatment [14]. The proposed mechanisms of action of different orally given probiotic strains are influences on mucosal immune cells, e.g., the IL-10 inducing effect of *Faecalibacterium prausnitzii* in dendritic cells (DCs) and subsequent modulation of regulatory T cells (Tregs). Furthermore, a mix of Clostridia strains stimulated mucosal Tregs, *Lactobacillus rhamnosus* had direct impacts on the mucosal barrier and *Bifidobacterium bifidum* changed the gut microbiota composition [15–18]. In IBS, different types of probiotics showed influences on fecal and mucosal microbiota composition as well as intestinal barrier function, but systematic investigation of the effects on different regions of the GI tract and the mucosal immune system is lacking [19–21]. Furthermore, *Bifidobacterium bifidum* has been shown to influence the level of fecal short-chain fatty acids (SCFAs) in humans, which are known to play an important role in mediating gastrointestinal homeostasis [18, 22]. However, data on clinical efficacy and mechanisms of action of synbiotics in IBS, especially regarding possible region-specific differences in the upper and lower GI tracts, remain to be scarce [23]. To elucidate which regions and levels of the intestinal ecosystem could be involved in mechanisms of action of synbiotics in IBS-D, we investigated the GI tract in ten patients with IBS-D before and after a 4-week treatment with an open-label oral synbiotic mixture (containing multiple species of probiotic strains and prebiotics including inulin, starch, and fructooligosaccharides). The effects of synbiotic treatment were determined by exploring mucosal immune cells (sampled in duodenum and ascending colon), SCFAs, and zonulin (a surrogate for the intestinal barrier function [24]), from stool and the gastrointestinal microbiota (from stool as well as from mucosal samples originating from stomach, duodenum, and colon).

## Methods

We investigated the effects of a 4-week oral synbiotic on ten IBS-D patients by comparing the following parameters pre- and post-treatment. Patients were examined by endoscopic evaluation of the upper and lower gastrointestinal tracts to obtain mucosal samples for FACS analysis and mucosal 16S rDNA analysis. Thereby, biopsies for FACS analysis were separately taken from the duodenum and ascending colon during the retraction of the endoscope and immediately processed. The colonic biopsies were obtained between the right-colonic flexure and the caecum. In addition, biopsies from the gastric corpus, duodenum, and ascending colon were obtained for mucosal microbiota analysis. Furthermore, analysis of fecal SCFAs, zonulin, and fecal 16S rDNA was performed.

### Patients and controls

IBS-D patients (according to S3 guidelines) for this study were recruited consecutively from the outpatient clinics of the Department of Internal Medicine, Division of Gastroenterology and Hepatology, Medical University of Graz [25]. The following inclusion criteria were applied: (1) symptomatic IBS patients according to current S3 guideline [25]; (2) age between 18 and 65 years; and (3) informed consent. Exclusion criteria were: (1) chronic inflammatory (IBD, celiac disease, and microscopic colitis were ruled out, and patients had the previous endoscopic evaluation and diagnostics), immune-, or neoplastic diseases; (2) recent application of immune-modifying medication; (3) pregnancy; and (4) alcohol or drug abuse. No patients received any new medications during the study period and no changes in medication dose were made during the study. No antibiotics were taken 4 weeks prior to study inclusion. All medications used by patients are systemically compiled in Table 1. All individuals included signed an informed consent prior to study inclusion. All protocols and informed consents were a priori waived by the local institutional review board (IRB number IRB00002556), vote 25-594 ex 12/13.

**Table 1:** Clinical and demographic data of ten patients with IBS-D included in the study

Number of patients (% female)	10 (50)
Age at endoscopy, years (median [Q1–Q3])	46 [37–53]
BMI (median [Q1–Q3])	23 [22–25]
IBS-SSS baseline (median [Q1–Q3])	236 [129–256]; IBS diagnosis > 75
Relevant comorbidities (number of patients)	Hypothyroidism (4)
	Iron deficiency (2)
	Depression (2)
	Bronchial asthma (1)
	Gastroesophageal reflux disease (1)
	Arterial hypertension (1)
	Osteopenia (1)
	Osteoporosis (1)
Co-medications (number of patients)	Thyroid hormones (4)
	Vitamin D3 (3)
	Proton pump inhibitor (2)
	Calcium (2)
	Antidepressant (2)
	H2-blocker (1)
	Selective estrogen receptor modulator (1)
	Beta blocker (1)
	Benzodiazepine (1)
	Atypical antipsychotic (1)
	Antacid (1)
	Antihypertensive (1)
	Spasmolytic (1)
	Prokinetic (1)
	Folate (1)
	Vitamin B complex (1)

*IBS-D* diarrhea-predominant irritable bowel syndrome, *BMI* body mass index, *IBS SSS* irritable bowel syndrome severity scoring system

### Study protocol and schedule

Patients were recruited from the outpatient department as mentioned. During a screening visit, inclusion and exclusion criteria were applied and physical status investigated. Patients fulfilling criteria signed an informed consent and were scheduled for the baseline study visit 1. At study visit 1, upper and lower GI tract endoscopy was performed and biopsies were taken, fecal samples and IBS-SSS were obtained, and synbiotic formulation was handed out. Patients recorded the oral administration of synbiotic mixture twice a day for 4 weeks. At study visit 2 (4 weeks later), all examinations were re-performed including endoscopy and obtaining of fecal samples and IBS-SSS.

### IBS-SSS

The German version of the validated IBS-SSS questionnaire was obtained from the Zentrum für klinische Ernährung (ZKES, Wollgrasweg 49b, 70599 Stuttgart, Germany) and used as described [26]. Symptoms were quantified prior and after 4 weeks of synbiotic therapy.

### Synbiotic formulation

Patients were given a 4-week course (twice a day) commercially available synbiotic mixture (OMNi-BiOTiC^®^ Stress Repair, Institut Allergosan, Graz) consisting of the following prebiotics corn starch, maltodextrin, inulin, fructooligosaccharides, potassium chloride, magnesium sulfate, mangan sulfate and enzymes as well as 7.5 × 10^9^ of each of the following probiotic bacterial strains: *Lactobacillus casei W56, Lactobacillus acidophilus W22, Lactobacillus paracasei W20, Lactobacillus salivarius W24, Lactobacillus plantarum W62, Lactococcus lactis W19, Bifidobacterium lactis W51* and *W52*, and *Bifidobacterium bifidum W23*.

### Mucosal specimens

Gastroduodeno- and ileocolonoscopy was performed with standard equipment (Olympus, Hamburg, Germany) in sedated subjects. Samples were obtained by forceps biopsy. Biopsies for FACS analysis were separately taken from the duodenum and ascending colon during the retraction of the endoscope and immediately processed. The colonic biopsies were obtained between the right-colonic flexure and the caecum. In addition, biopsies from the gastric corpus, duodenum, and ascending colon were obtained for mucosal microbiota analysis.

### Isolation of lamina propria mononuclear cells

Mucosal biopsy specimens were obtained separately from the duodenum and ascending colon and immediately preserved in chilled RPMI medium (Sigma; supplemented with penicillin, streptomycin, and amphotericin). Biopsies were washed once with calcium- and magnesium-free HBSS (Life Technologies, Vienna, Austria) and then incubated in calcium- and magnesium-free HBSS containing 1 mM DTT and 5 mM EDTA at 37 °C for 20 min with gentle agitation to remove mucus and epithelial cells. Following a brief wash with calcium- and magnesium-free HBSS, tissue was digested with 1 mg/ml Collagenase A (Roche, Basel, Switzerland) and 5 units/ml DNase I (Roche, Basel, Switzerland) in HBSS at 37 °C for 60 min on a shaker and mechanically disrupted by gentle pipetting. Complete dissociation was verified by visual inspection. After passing through a 70 µm cell strainer, the released cells were washed twice with RPMI complete medium (containing 10% FCS and 1% penicillin/streptomycin) and finally re-suspended in RPMI complete medium. The cell suspension was kept on ice until further analysis.

### Flow cytometry

FACS analysis was performed as previously described [27–29]. Briefly, the cell suspension was washed once with staining buffer (PBS containing 3% FCS and 2 mM EDTA) and the cells were stained in 100 µl staining buffer for 20 min at room temperature in the dark. For enumeration of lamina propria dendritic cells, directly labeled monoclonal antibodies for the following markers were used: lin (lineage) 1-FITC (CD3, CD14, CD16, CD19, CD20, CD56, and CD34), HLA-DR-PerCP-Cy5.5, CD11c-APC, and CD103-PE. LPDCs were identified as lin1-/HLA-DR^+^ cells. For determination of Tregs, anti-CD3-APC-Cy7, anti-CD4-V450, anti-CD8-FITC, anti-CD25-PE, and anti-CD127-Alexa Fluor 647 antibodies were used. With the exception of CD103-PE (eBioscience, San Diego, USA), all antibodies were purchased from BD Bioscience (San Jose, USA). FMO (fluorescence-minus-one) controls were employed to set the boundaries for gating of positively stained cells. After the staining reaction, the cells were washed once with staining buffer and re-suspended in 100 µl staining buffer. For the exclusion of dead cells, propidium iodide (PI) was added to the samples immediately prior to acquisition on an LSR II (BD Bioscience, San Jose, USA) flow cytometer. The data files were analyzed using FlowJo (FlowJo, LLC) software.

### Isolation of total genomics DNA, 16S library preparation, and Illumina sequencing

Stool and mucosal samples were stored at − 80 °C and used for total DNA isolation combining mechanical and enzymatic lysis with the MagnaPure LC DNA Isolation Kit III (Bacteria, Fungi) (Roche, Mannheim, Germany) according to manufacturer’s instructions as described [30]. Modifications were made for stool and mucosal specimens. Briefly, stool samples were homogenized in 500 µl PBS and 250 µl of the suspension was mixed with 250 µl of bacterial lysis buffer and further transferred to Magna Lyser green bead tubes (Roche, Mannheim, Germany). Mechanical lysis was two times performed at 6500 rpm in a MagNA Lyser Instrument (Roche, Mannheim, Germany). Mucosal specimens were prepared with bead beating for four times at 6500 rpm in 500 µl lysis buffer and enzymatic lysis samples were mixed with 25 µl lysozyme (100 mg/ml) and incubated at 37 °C for 30 min. Afterwards, samples were mixed with 30 µl Proteinase K and stool samples were incubated at 65 °C for 1 h. Mucosal specimens were incubated overnight at 65 °C. Enzymes were heat inactivated at 95 °C for 10 min and further steps were performed according to Magna Pure DNA isolation kit III (Bacteria, Fungi) manufacturer’s instruction. 250 µl of mucosal specimens and 100 µl of stool samples were taken for DNA purification that was eluted in 100 µl. For target specific PCR amplification, the primers 27f (AGAGTTTGATCCTGGCTCAG) and 357r (CTGCTGCCTYCCGTA) were taken as described by Baker et al. [31] and synthesized at Eurofins (MWG, Ebersberg, Germany). Then, 5 µl of total DNA from mucosal sample and 2 µl from stool sample extracts were taken for a 25 µl PCR reactions as described [30]. Triplicates were pooled and amplification was verified using a 1% agarose gel. The sequencing library was amplified, quantified, and sequenced on a MiSeqII desktop sequencer (Illumina, Eindhoven, The Netherlands) as described previously [30]. Version 3.600 cycles chemistry (Illumina, Eindhoven, The Netherlands) was taken according to manufacturer‘s instructions to run the 6 pM library with 20% PhiX (Illumina, Eindhoven, The Netherlands). FASTQ files were then further taken to perform data analysis.

### Microbiota analysis and statistical methods

Quality filtering and analysis of raw 16S rRNA gene sequence data (hypervariable region V1–V2) was performed with mothur (version 1.22.0) according to the recommended standard operating procedure of mothur for Illumina MiSeq data (https://www.mothur.org/wiki/MiSeq_SOP, accessed June 2016) with additional removal of singletons (default settings and parameters were used, if not specified otherwise) [32, 33]. Briefly: paired reads were merged using mothur’s make.contigs command, whereby reads less than 200 bps were filtered out of the data set. In addition, sequences containing ambiguous bases or more than eight homopolymeres were removed together with chimeric sequences or sequences outside of the core alignment with the SILVA reverence database (version 119). Furthermore, noisy sequences were identified using pre.cluster and finally deleted from the data set. Remaining pre-processed and filtered sequences were clustered by mothur’s de novo OTU-picking strategy into OTUs at a distance of 0.03. Finally, taxonomic classification was assigned using the RDP Bayesian classifier (version 2.10.1, trainingsset 10/29.10.2014) with default settings and a classification confidence cutoff of 80% [34]. Subsequent OTU-based microbiota analyses were performed in QIIME (version 1.8.0), including core.diversity analysis with rarefaction to a sampling depth of 9.538 reads per sample for all four locations (COR = corpus, COL = colon, FEC = feces, and DUO = duodenum) [35]. Unweighted UniFrac distance metrics as measure of between-sample (beta) diversity was calculated and applied for principal coordinates analysis (PCoA) to visualize patterns of diversity [36]. Within-samples (alpha) diversity was calculated using four different measures (1) observed species, (2) ChaoI Index, (3) Shannon Index, and (4) Faith’s phylogenetic diversity [37, 38]. Statistical significant differences between sample (alpha) diversity were assessed by a nonparametric two-sample *t* test (*p* values were determined by Monte Carlo permutations. Calculations are based on the greatest rarefaction depth. Bonferroni correction was used to account for multiple comparisons). Differences in taxonomic microbiota compositions (differentially abundant features/genera) within the four locations and between treatments were determined using linear discriminant effect size analysis (LEfSe) on the filtered data sets at species level. If not otherwise specified *p* values below 0.05 were considered as statistically significant [38].

### Availability of data and materials

The sequence data supporting the results of this article are available in the European Bioinformatics Institute Sequence Read Archive under accession number PRJEB19253.

### Extract preparation from specimens for multiplex cytokine assay

Colonic and duodenum specimens were obtained in cold RPMI1640 medium supplemented with penicillin and streptomycin. Samples were transferred to cryotubes, snap frozen, and stored in liquid nitrogen until sample preparation. All samples were then individually thawed on ice and immediately disrupted in 300 µl extraction buffer for 2 min on ice with a pellet pestle (Kimble Kontes, USA). Extraction buffer comprised DPBS (Dulbecco’s phosphate buffered saline without calcium and magnesium, Lonza) and EDTA-free protease inhibitors (cOmplete mini, Roche). After further disrupting them mechanically by pipetting, biopsies were passed through a 70 µm cell strainer. All samples were then incubated on ice for 5 min. Finally, supernatants were obtained by centrifugation at 10,000×*g* for 10 min at 4 °C, snap frozen in liquid nitrogen, and stored at − 80 °C until analysis. Cytokine analysis included IL-1β, IL-6, IL-10, IL-12p40, IL-12p70, IL-17A, IL-23, and TNF-α. Multiplex immunoassay kits (ProcartaPlex) used for analysis were obtained from eBioscience and were run according to manufacturer’s instructions using magnetic beads. Standards for each cytokine were assayed in duplicates to generate standard curves using the reference concentrations as provided by the manufacturer. All samples were individually thawed on ice and wash steps were performed using a hand-held magnetic block. Data were obtained on a validated and calibrated Bio-Plex 200 system (Bio-Rad) and analyzed with Bio-Plex Manager 6.1 software (Bio-Rad). BCA Protein Assay (Pierce) was used to determine total protein concentration and to normalize cytokine concentrations for each sample.

### GC–EI/MS of short-chain fatty acids

SCFAs (acetic acid, propionic acid, iso-butyric acid, butyric acid, iso-valeric acid, and valeric acid) were extracted from stool frozen at − 80 °C. SCFA concentrations were measured by a GC–MS equipped with a PEG DB-WAXetr. (30 m; 0.25 mm ID; and 0.25 µm film) column. SCFA were extracted from feces by sequential addition of 1 ml phosphoric acid (0.5%) and 1 ml methyl-*tert*-butyl-ether, 10 min shaking, 10 min centrifugation, and removal of the upper organic layer. Before extraction 100 nmol of d-acetic acid, d-propionic acid, d-butyric acid, and d-valeric acid were added as internal standards. Calibration curves by stable isotope dilution were performed from 0.1 to 2.000 µM for acetic acid, propionic acid, iso-butyric acid, butyric acid, iso-valeric acid, and valeric acid. A 7890B/5977A MSD GC–MS (Agilent, Waldbronn, Germany) equipped with a PEG DB-WAXetr. (30 m; 0.25 mm ID; and 0.25 µm film) column was used. Helium was used as carrier gas at 1.3 ml/min in splitless mode at 250 °C injector temperature. The initial oven temperature of 60 °C was held for 2 min, and then, the temperature first was ramped up to 150 °C at a rate of 15°C/min. This was followed by a ramp of 5°C/min up to 170 °C and 20°C/min up to 250 °C, where the temperature was held for another 2 min. The mass spectrometer was run in electron impact (EI) mode, where the SCFAs were detected in SIM mode on *m*/*z* 60, 63, 73, 74, 76, 79, and 80. The source temperature was set to 250 °C and the transfer line temperature was 280 °C. Data analysis was performed by Mass Hunter (Agilent, Waldbronn, Germany).

### Zonulin

A ready-to-use solid-phase sandwich ELISA (Immundiagnostik AG, Bensheim, Germany) was used to detect zonulin (zonulin Stool ELISA) in fecal samples. The tests were performed according to the manufacturer’s instructions. For stool sampling, the Stool Sample Application System (Immundiagnostik AG, Bensheim, Germany) was used according to the manufacturer’s manual [39].

### Statistics

Statistical analyses were carried out using SPSS 22 (IBM^®^ Corporation USA) and GraphPad Prism^®^ 6.0 (GraphPad Software, Inc., USA). Values are presented as number (%) or median [interquartile range]. For the comparison of categorical variables, we applied Fisher’s exact test. Group differences of continuous variables were determined by Mann–Whitney *U* test or *t* test depending on the data distribution (non-gaussian or gaussian). Boxplots are depicted according to Tukey.

## Results

### Patients

Ten patients with IBS-D consented to the study. Their median age was 46 [37–53] years (median [Q1–Q3]) of which 5/10 were women. Four patients had no relevant medical comorbidities, and those of the remaining six patients are compiled in Table 1.

### Effect of synbiotic treatment on mucosal immune cell lineages and mucosal cytokine levels in different regions of the gastrointestinal tract

**Fig. 1:**
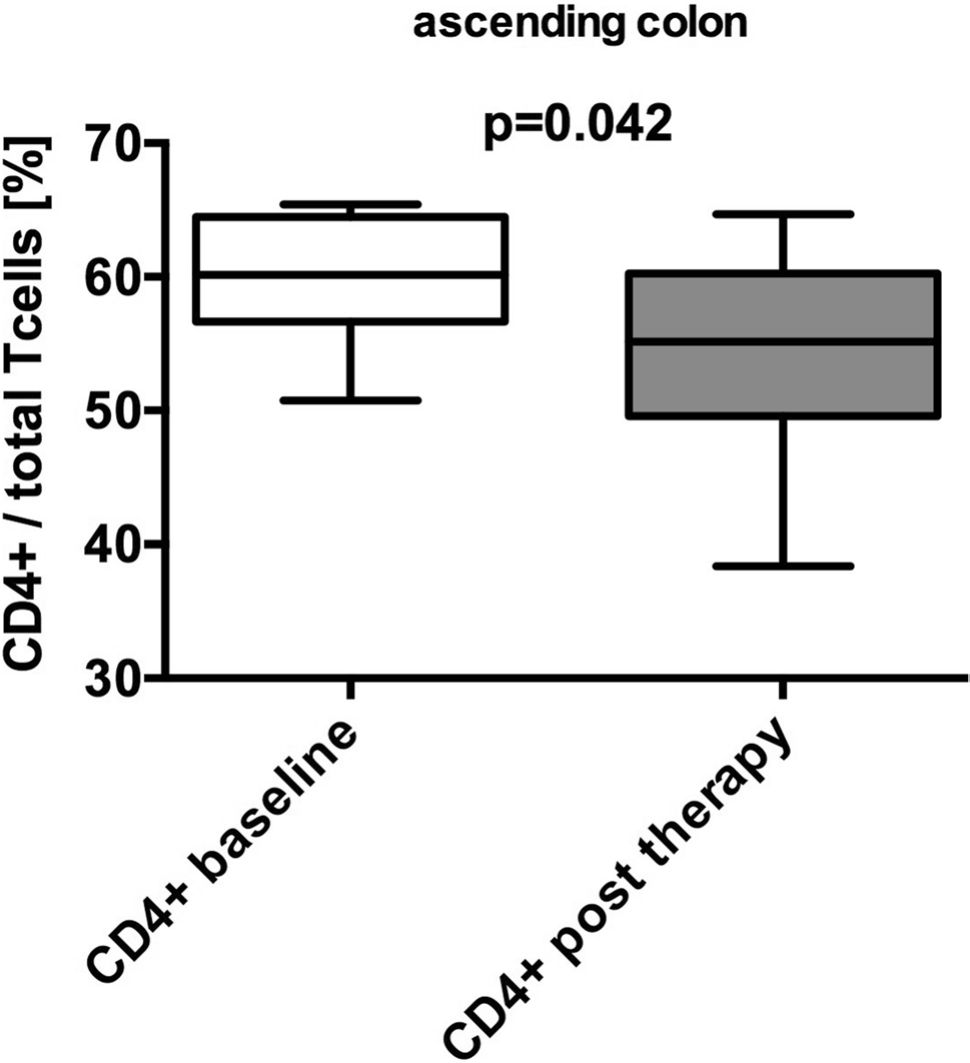
Reduction of mucosal CD4^+^ T cells after synbiotic treatment. A significant reduction of CD4^+^ T cells (%), sampled from mucosa specimens from the ascending colon, was found after (grey) synbiotic treatment compared to baseline (white) (60 [57–65] vs. 55 [50–60], *p* = 0.042) (median [Q1–Q3]; Mann–Whitney *U* test or *t* test to compare non-gaussian and gaussian variables. Boxplots according to Tukey)

We performed FACS analyses of human mucosal specimens (biopsies from the duodenum, ascending colon) after collection of tissue samples during endoscopy before and after synbiotic treatment. Mucosal T- and dendritic cell subsets were then characterized by FACS analysis. Mucosal immune cell results are systematically listed in suppl. Table 1. In the ascending colon, a significant reduction of mucosal CD4^+^ T cells (60 [57–65] vs. 55 [50–60] %, *p* = 0.042) was observed after synbiotic treatment (Fig. 1). Furthermore, double-negative T cells (CD3^−^ CD4^−^ T cells) showed a trend towards elevation (10 [9–12] vs. 13 [8–25] %, *p* = 0.078) after synbiotic treatment in the ascending colon. Dendritic cells could not be isolated from the duodenal mucosa. No synbiotic treatment-associated changes of DCs (total, CD11c^+^, or CD103^+^) were found in the ascending colon. No significant changes of T-cell subsets were found in the duodenum. Mucosal extract cytokine concentration was measured in mucosal specimens of the duodenum and ascending colon and results are systematically compiled in Suppl. Table 2. A trend towards higher concentration of tumor necrosis factor alpha (TNF-alpha) was evident after synbiotic treatment in the ascending colon (0.2 [0–0.4] vs. 0.6 [0–1.1] pg/ml, *p* = 0.0547). Other cytokines were not affected by synbiotic treatment.

### Increased phylogenetic diversity in the upper but not lower gastrointestinal tract after synbiotic treatment

**Fig. 2:**
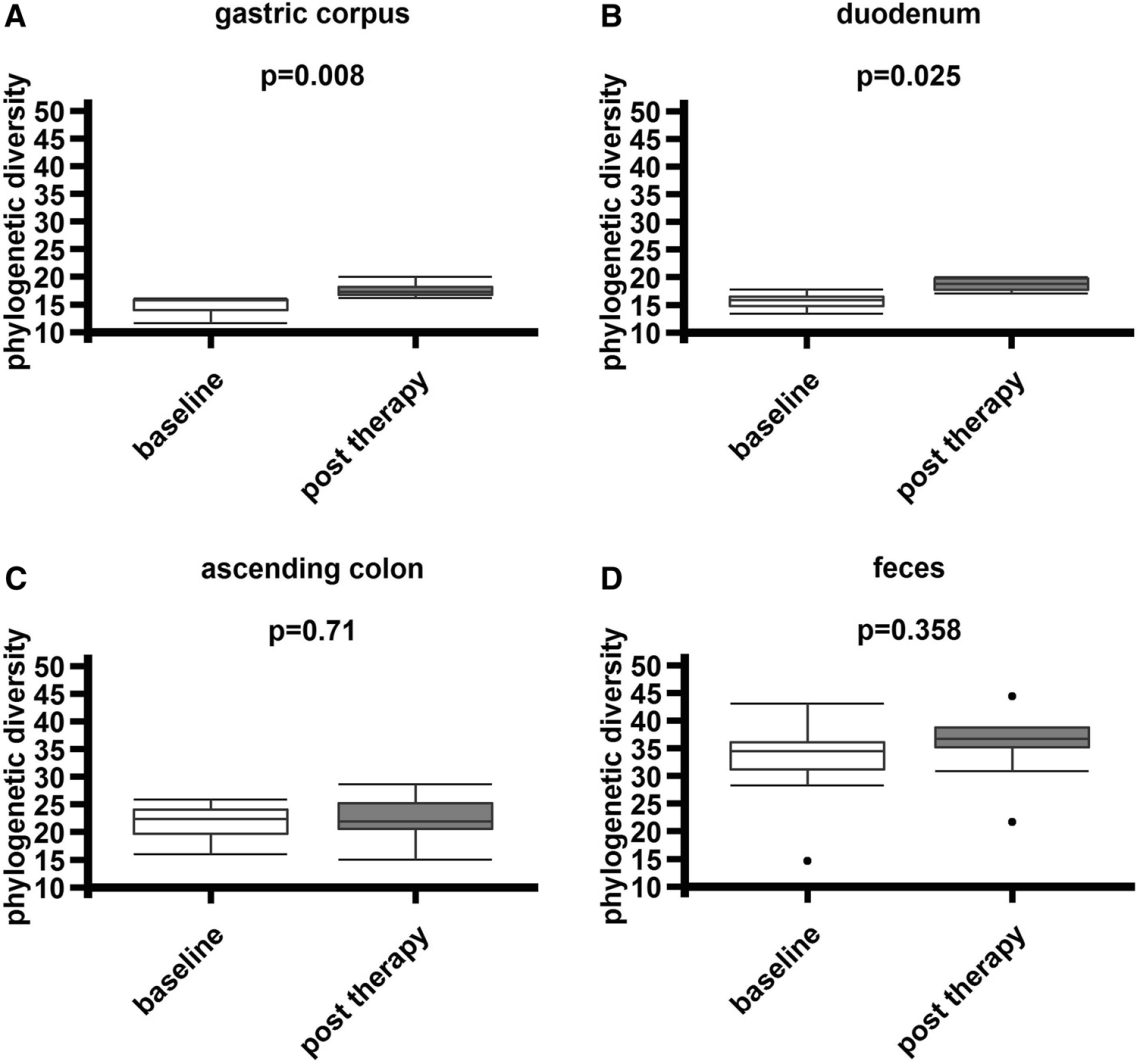
Increased phylogenetic diversity in the upper- but not lower gastrointestinal tract after synbiotic treatment. **a, b** Phylogenetic diversity (Faith) increased in specimens from the gastric corpus (*p* = 0.008) and duodenum (*p* = 0.025). **c, d** No differences in colonic samples (*p* = 0.710) and feces (*p* = 0.358) were evident (Faith’s phylogenetic diversity, based on UniFrac phylogenetic distance, nonparametric two-sample *t* test to determine *p* values using Monte Carlo permutations, post-error correction Bonferroni)

To further elucidate the effects of synbiotic treatment on different regions of the gastrointestinal tract (gastric corpus, duodenum, ascending colon, feces), richness (observed species) phylogenetic diversity (Faith’s phylogenetic diversity), and Shannon Diversity Index in mucosal and fecal samples were determined. Significant elevations in phylogenetic diversity could be observed in mucosal samples of the gastric corpus (*p* = 0.008) and duodenum (*p* = 0.025) as well as richness in the duodenum (*p* = 0.011). No differences were found in colonic (*p* = 0.710) and fecal samples (*p* = 0.358, Fig. 2). All results are shown in suppl. Table 3.

### Microbial abundances associated to synbiotic treatment

**Fig. 3:**
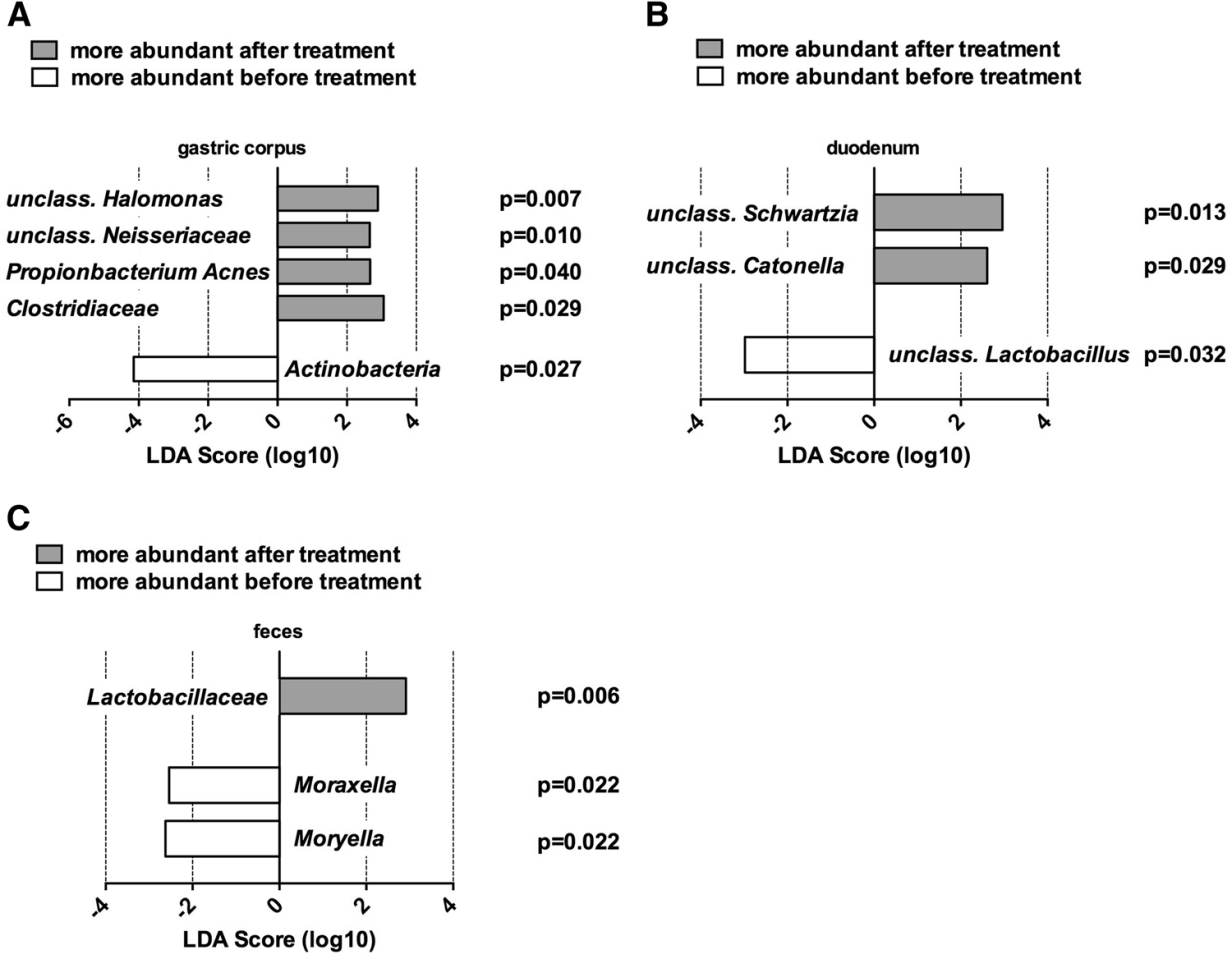
Linear discriminant analysis of mucosal and fecal samples pre- and post-synbiotic treatment. Linear discriminant analysis (LDA) was generated with LEfSe. **a** Mucosal specimens of the gastric corpus showed elevated relative abundances of unclassified *Halomonas* (*p* = 0.007), unclassified *Neisseriaceae* (*p* = 0.010), *Propionibacterium Acnes* (*p* = 0.040), and *Clostridiaceae* (*p* = 0.029) together with a reduction of Actinobacteria (*p* = 0.027) after synbiotic treatment. **b** Mucosal specimens of the duodenum depicted increased unclassified *Schwartzia* (*p* = 0.013) and *Catonella* (*p* = 0.029) as well as a reduction of an unclassified Lactobacillus after synbiotic treatment. **c** Fecal samples showed increased *Lactobacillaceae* (*p* = 0.006) as well as diminished *Moraxella* (*p* = 0.022) and *Moryella* (*p* = 0.022) after synbiotic treatment

Bacterial community profiling was performed to investigate upper and lower gastrointestinal mucosal specimens and fecal samples. Thereby, mucosal specimens of the gastric corpus revealed raised relative abundances of unclassified *Halomonas* (*p* = 0.007), unclassified *Neisseriaceae* (*p* = 0.010), *Propionibacterium acnes* (*p* = 0.040), and *Clostridiaceae* (*p* = 0.029), whereas *Actinobacteria* (*p* = 0.027) were reduced after synbiotic treatment. In duodenal specimens, elevated unclassified *Schwartzia* (*p* = 0.013) and *Catonella* (*p* = 0.029) in addition to a reduction of an unclassified *Lactobacillus* after synbiotic treatment was evident. No taxa showing significantly different relative abundances before and after treatment were observed in colonic mucosal specimens. No probiotic strain of the synbiotic mixture could colonize the gut. A higher abundance of unclassified *Lactobacillaceae* (*p* = 0.006) could be found in fecal samples after synbiotic treatment. *Moraxella* (*p* = 0.022) and *Moryella* (*p* = 0.022) were reduced after treatment in fecal samples (Fig. 3).

### Increased SCFA levels and reduction of fecal zonulin in fecal samples after synbiotic treatment

**Fig. 4:**
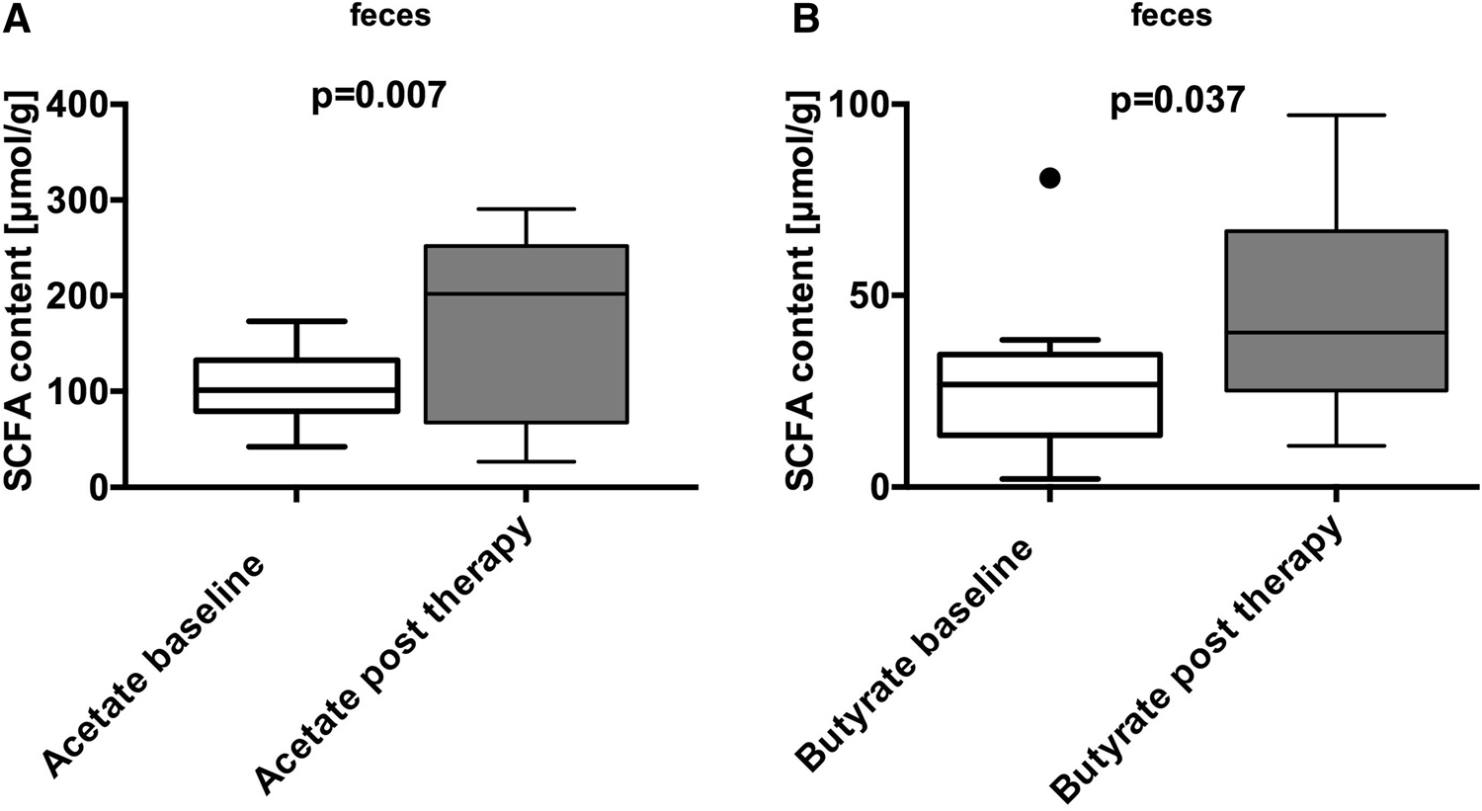
Elevated SCFA levels in fecal samples of patients after synbiotic treatment. Fecal samples were analyzed by HPLC. A significant elevation in fecal acetate and butyrate levels (µmol/g) before (white) vs. after synbiotic treatment (grey) were found. Acetate (101 [79–133] vs. 202 [68–252]; *p* = 0.007) and butyrate content (27 [14–35] vs. 40 [25–67]; *p* = 0.037) (median [Q1–Q3]; Mann–Whitney *U* test or *t* test to compare non-gaussian and gaussian variables. Boxplots according to Tukey)

We examined levels of SCFAs in the fecal samples of patients by high-performance liquid chromatography (HPLC). Significant increases in acetate (101 [79–133] vs. 202 [68–252] µmol/g; *p* = 0.007) and butyrate (27 [14–35] vs. 40 [25–67] µmol/g; *p* = 0.037) levels after treatment were found (Fig. 4), whereas propionate, iso-butyrate, iso-valerate, and valerate levels remained unaltered. Fecal concentrations of zonulin were measured using competitive ELISA in fecal samples. Zonulin concentrations (ng/ml) decreased significantly after synbiotic treatment (67 [38–92] vs. 36 [20–48] ng/ml; *p* = 0.035; Suppl. Figure 1).

### Reduction of the symptom severity after synbiotic treatment

The IBS-SSS (irritable bowel syndrome–severity scoring system) was used to define clinical effects of synbiotic treatment. Patients showed significant improvements in IBS-SSS. (237 [129–256] vs. 54 [12–158]; *p* = 0.002; Suppl. Figure 2).

## Discussion

In the present pilot study, we conducted a systematic investigation of the GI tract before and after a 4-week treatment with an open-label oral synbiotic mixture in patients with IBS-D. We thereby assessed the mucosal immune system and microbiota, fecal microbiota and SCFAs, clinical IBS activity, and mucosal permeability. Our data suggest varying GI effects of oral synbiotic treatment in IBS-D patients. The most significant changes were seen in mucosal microbiota biodiversity of the upper GI tract, accompanied with effects on mucosal immune cell subsets, microbial metabolic activity and small intestinal mucosal barrier function reflected by zonulin levels [40].

First, synbiotic treatment influenced mucosal phylogenetic bacterial diversity in the upper but not the lower gastrointestinal tract showing pronounced increases of phylogenetic diversity of the microbiota in gastric and duodenal mucosa, whereas diversity remained unaffected in colon and feces. In addition, *Lactobacillaceae*, belonging to the synbiotic mixture, were elevated only in fecal samples, but were not found in any mucosal sample. Fecal samples depicted a notably reduction of *Moraxella* and *Moryella*, but the importance of this observations is unclear. The observed elevations of phylogenetic diversity in gastric and duodenal specimens were not chaperoned by increases of the orally administered probiotic strains used for treatment, which is a known phenomenon in probiotic therapy. Nevertheless, oral probiotics might have a catalyzing effect on mucosal richness [13]. Although changes in microbial composition of the rectal mucosa have been described after probiotic therapy of IBS patients, we herein show a more comprehensive investigation of how synbiotic preparation affects the intestinal ecosystem in different regions of the GI tract [19]. Furthermore, our results suggest that synbiotic effects on microbial composition in IBS-D are preferentially observed in the upper gastrointestinal tract. Whether the observed treatment-induced changes are due to the probiotic or prebiotic ingredients of the administered mixture remain unclear, since prebiotics are also known to influence intestinal microbial composition in humans [41]. Taken together, this study gives a comprehensive oversight of 16S rRNA changes in different habitats and locations of the gastrointestinal tract induced by an oral synbiotic. Specifically, we show for the first time that the focus of mucosal microbial responses to oral synbiotic therapy is located in the upper but not the lower gastrointestinal tract.

Second, oral synbiotic treatment significantly reduced the number of CD4^+^ T cells in the ascending colon. Increased mucosal inflammation is associated with IBS-D pathogenesis and disease activity. Moreover, elevated numbers of T cells have been shown in jejunal and colonic specimen of IBS-D patients vs. controls. However, these cells were not differentiated further, which could be of relevance, because naïve T cells can develop into pro-as well as anti-inflammatory T cells [5, 42, 43]. The treatment-induced reduction of colonic CD4^+^ T cells in our study is likely to lead to an alteration of the colonic inflammatory status. In conjunction with the observed trend towards an induction of double-negative (CD4^−^ CD8^−^) T cells, for which Treg-like anti-inflammatory properties (suppression of CD8^+^ and CD4^+^ T cells, B cells and DCs) have been proposed, one could hypothesize an anti-inflammatory effect [44]. It is one of the limitations of this study that we neither differentiated further CD4^+^ T cells into pro- and anti-inflammatory subsets. It is worth noting that T cells in the upper GI tract, where the microbial changes were most pronounced, remained unaffected. In addition, neither mucosal regulatory T cells nor DCs could be shown to be affected by synbiotics in any compartment. In sum, the reduction of mucosal CD4^+^ T cells accompanied by enhanced double-negative T cells in the ascending colon could represent an anti-inflammatory modulation of the colonic mucosa, but this needs further confirmation.

Third, we show that synbiotic treatment elevated fecal levels of the SCFAs acetate and butyrate. The synbiotic mixture could stimulate acetate and butyrate levels by its prebiotic or probiotic components, as prebiotics are known to stimulate, e.g., butyrate production in humans and *Lactobacillaceae* (elevated in fecal samples after treatment) can mediate butyrate production [41, 45]. Furthermore, probiotic bacteria are metabolically active depending on the host microbiota and initial fecal butyrate level in healthy individuals. Thereby, the impact of probiotics on fecal butyrate level was highest in individuals with low butyrate concentration, as observed in our study participants, before treatment [18, 22, 46]. It was previously demonstrated that IBS patients show decreased fecal butyrate levels, which is of importance, since acetate and butyrate are known to induce mucosal Tregs and regulatory DCs [22, 47]. We hypothesize that the treatment-associated elevation of acetate and butyrate could be involved in the observed mucosal T-cell changes and the clinical responses to the study preparation.

Intestinal SCFAs such as butyrate are also known to stabilize intestinal barrier function even after chemical disruption and might, therefore, be of importance in prophylaxis and treatment of gut leakiness [48, 49]. IBS-D patients exert intestinal permeability disruptions and abnormalities in their epithelial barrier function, as shown in the previous studies [8, 10]. In this study, fecal zonulin concentration, a surrogate marker for the intestinal barrier, was significantly reduced by synbiotics. This reflects a possible treatment effect on intestinal barrier function in our IBS-D cohort. The stabilization of the epithelial barrier could subsequently influence mucosal inflammation in IBS-D [9, 10] and contribute to a significantly lower symptom severity score after treatment. In sum, synbiotic therapy elevated fecal acetate and butyrate levels accompanied by a reduction of fecal zonulin and symptom severity. These observations indicate possible effects of synbiotic therapy on microbiota metabolism and intestinal barrier function.

In conclusion, the present pilot study indicates possible mechanisms of action of oral synbiotic therapy in IBS-D. The observed effects comprise an elevation of mucosal microbial diversity, the elevation of colonic CD4^+^ T cells, the elevation of fecal acetate and butyrate levels and a decrease of fecal zonulin, and a surrogate of intestinal barrier function. The potency of oral synbiotics in the treatment of IBS-D is underlined by the clinical response observed in this study by decreased IBS-SSS counts. The major limitations of the study are the lack of a placebo-controlled double-blinded design and the limited number of participants, which are limitations owing to the design as a pilot study. This in turn does not allow any causal interpretation of the results. Nevertheless, our study provides a systematic analysis of the possible effects of oral synbiotic therapy in IBS-D patients and gives the rationale for a larger-scale randomized trial.

## Electronic supplementary material

Below is the link to the electronic supplementary material.

Supplementary material 1 (JPG 113 KB)

Supplementary material 2 (JPG 90 KB)

Supplementary material 3 (PDF 251 KB)

Supplementary material 4 (PDF 20 KB)

Supplementary material 5 (PDF 140 KB)
