# Supplementary figures and images for: Effects of an oral synbiotic on the gastrointestinal immune system and microbiota in patients with diarrhea-predominant irritable bowel syndrome
Source: Eur J Nutr. 2018 Sep 24;58(7):2767–78. doi: 10.1007/s00394-018-1826-7 (PMC6768888; doi:10.1007/s00394-018-1826-7)

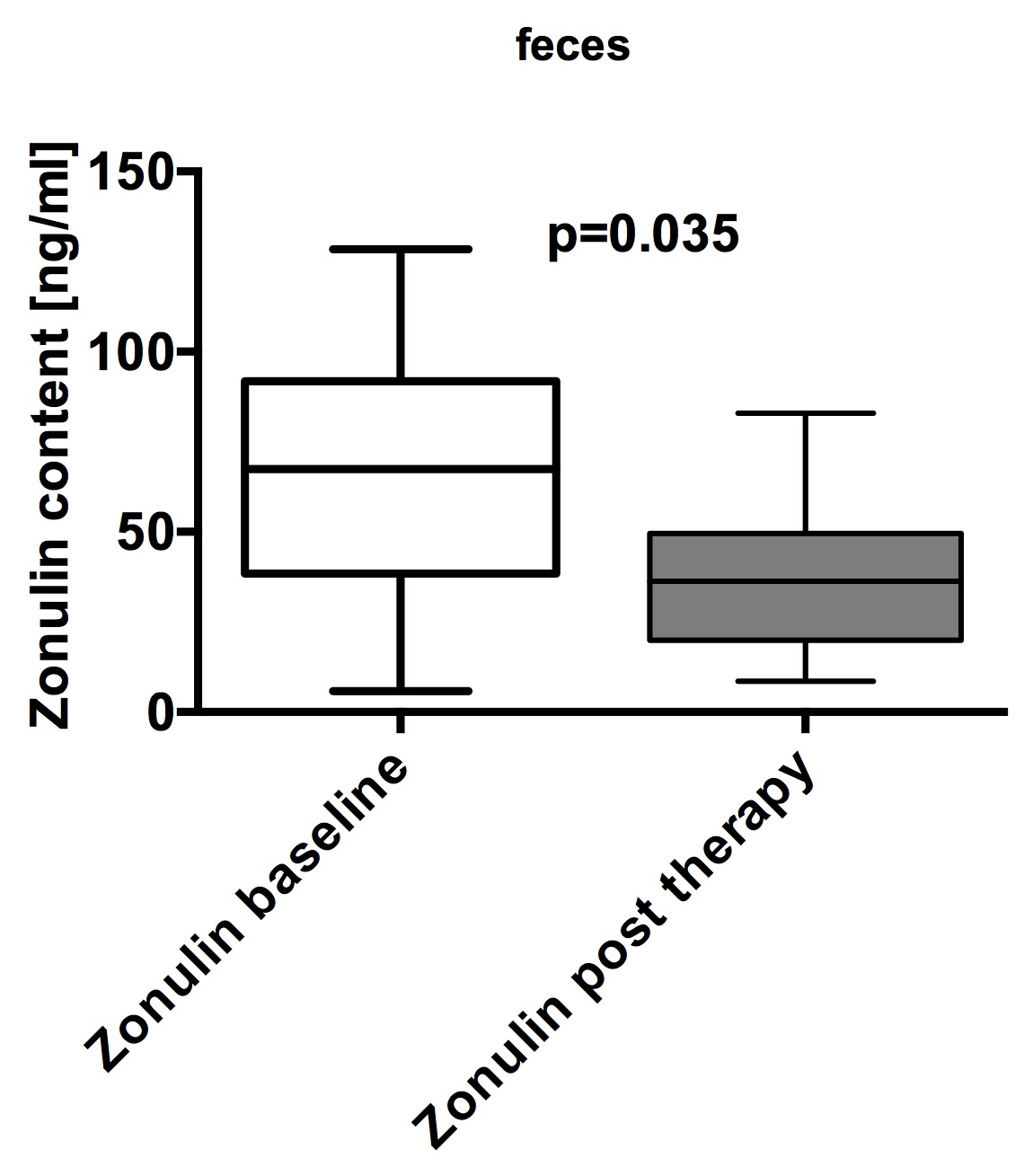

Supplement: Supplementary file 1 — Supplementary material 1 (JPG 113 KB) [file 394_2018_1826_MOESM1_ESM.jpg]

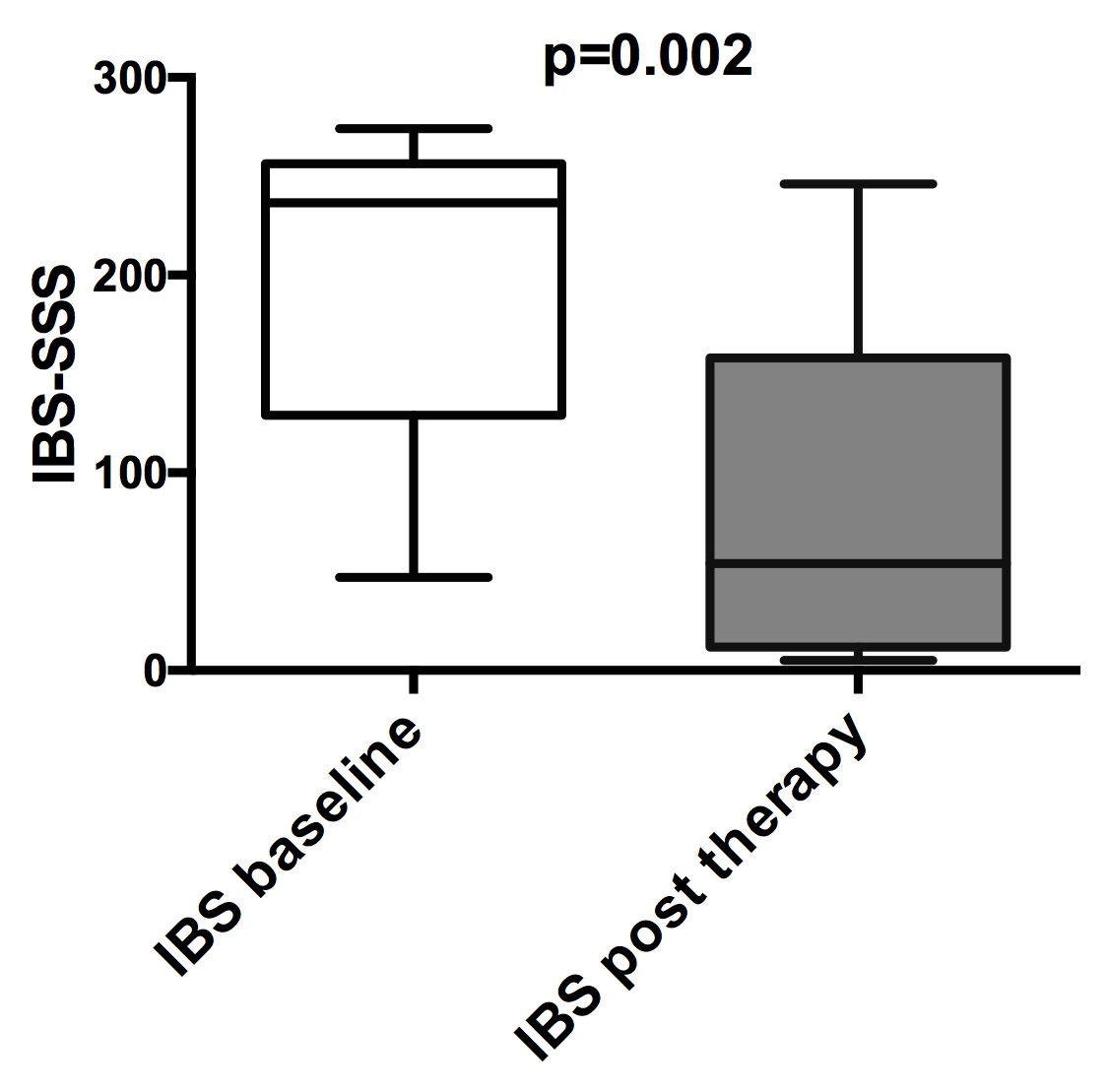

Supplement: Supplementary file 2 — Supplementary material 2 (JPG 90 KB) [file 394_2018_1826_MOESM2_ESM.jpg]
