# Supplementary material for: Effects of an oral synbiotic on the gastrointestinal immune system and microbiota in patients with diarrhea-predominant irritable bowel syndrome
Source: Eur J Nutr. 2018 Sep 24;58(7):2767–78. doi: 10.1007/s00394-018-1826-7 (PMC6768888; doi:10.1007/s00394-018-1826-7)
Supplement: Supplementary file 3 — Supplementary material 3 (PDF 251 KB) [file 394_2018_1826_MOESM3_ESM.pdf]

Suppl. Table 1. Mucosal immune cells

l.c.[a], living cells from the lymphocyte gate; l.c.[b], living cells of the lympho-monocyte gate; ns: not significant

	cell type	function	unit	median pre	median post	p
Duodenum						
	CD3+	T cell	[% l.c.[a]]	10.7	8.9	ns
	CD3+4+	T helper	[% CD3+]	19.4	23.8	ns
	CD3+8+	cytotoxic T lymphocyte	[% CD3+]	29.7	20.7	ns
	CD3+4+25+127-	Treg	[% CD4+]	1.2	1.5	ns
	CD3+4+8+	double-positive T cell	[% CD3+]	6.6	8.1	ns
	CD3+4-8-	double-negative T cell	[% CD3+]	22.9	23.4	ns
	CD3+56+16+	natural killer T cell	[% CD3+]	11.0	13.1	ns
	CD3-56+16+	natural killer cell	[% l.c.[a]]	4.5	3.0	ns
Proximal Colon						
	CD3+	T cell	[% l.c.[a]]	20.3	20.7	ns
	CD3+4+	T helper	[% CD3+]	59.1	54.7	0.042
	CD3+8+	cytotoxic T lymphocyte	[% CD3+]	21.8	18.8	ns
	CD3+4+25+127-	Treg	[% CD4+]	6.0	6.2	ns
	CD3+4+8+	double-positive T cell	[% CD3+]	1.9	2.3	ns
	CD3+4-8-	double-negative T cell	[% CD3+]	10.9	16.2	ns
	CD3+56+16+	natural killer T cell	[% CD3+]	2.6	2.0	ns
	CD3-56+16+	natural killer cell	[% l.c.[a]]	3.3	2.7	ns
	CD3-14-16-19-20-56-34-HLA-DR+	dendritic cell	[‰ l.c.[b]]	2.6	2.6	ns
	CD3-14-16-19-20-56-34-HLA-DR+103+	regulatory dendritic cell	[‰ l.c.[b]]	0.7	0.6	ns
	CD3-14-16-19-20-56-34-HLA-DR+11c+	11c+ dendritic cell	[‰ l.c.[b]]	2.4	2.3	ns
