# Supplementary material for: Effects of an oral synbiotic on the gastrointestinal immune system and microbiota in patients with diarrhea-predominant irritable bowel syndrome
Source: Eur J Nutr. 2018 Sep 24;58(7):2767–78. doi: 10.1007/s00394-018-1826-7 (PMC6768888; doi:10.1007/s00394-018-1826-7)
Supplement: Supplementary file 4 — Supplementary material 4 (PDF 20 KB) [file 394_2018_1826_MOESM4_ESM.pdf]

Suppl. Table 2. Mucosal cytokine levels
ns: not significant

	**cytokine type**	**unit**	**median pre**	**median post**	**p**
Duodenum					
	TNF alpha	[pg/ml]	2.4	2.8	ns
	IL12p40	[pg/ml]	6.7	6.8	ns
	IL12p70	[pg/ml]	1.4	1.3	ns
	IL6	[pg/ml]	10.3	9.4	ns
	IL23	[pg/ml]	0.3	4.2	ns
	IL17	[pg/ml]	0.5	0.7	ns
	IL10	[pg/ml]	0.2	0.3	ns
	IL1beta	[pg/ml]	3.3	3.0	ns
Colon					
	TNF alpha	[pg/ml]	0.2	0.6	0.0547
	IL12p40	[pg/ml]	11.2	5.9	ns
	IL12p70	[pg/ml]	0.6	0.8	ns
	IL6	[pg/ml]	0.0	0.0	ns
	IL23	[pg/ml]	0.0	0.0	ns
	IL17	[pg/ml]	0.0	0.0	ns
	IL10	[pg/ml]	0.1	0.1	ns
	IL1beta	[pg/ml]	4.2	3.5	ns
