# Supplementary material for: Effects of an oral synbiotic on the gastrointestinal immune system and microbiota in patients with diarrhea-predominant irritable bowel syndrome
Source: Eur J Nutr. 2018 Sep 24;58(7):2767–78. doi: 10.1007/s00394-018-1826-7 (PMC6768888; doi:10.1007/s00394-018-1826-7)
Supplement: Supplementary file 5 — Supplementary material 5 (PDF 140 KB) [file 394_2018_1826_MOESM5_ESM.pdf]

Suppl. Table 3. Mucosal Microbial Diversity depicted by using Phylogenetic Diversity, Observed Species and Shannon Diversity

PD: Phylogenetic diversity; ns: not

**Region**	PD	Observed Species	Shannon Diversity Index
gastric corpus	0.008	ns	ns
duodenum	0.003	0.011	ns
proximal colon	ns	ns	ns
feces	ns	ns	ns
